# The X-Rays in Exophthalmic Goitre

**Published:** 1908-08-29

**Authors:** 


### THE X-RAYS IN EXOPHTHALMIC GOITRE.

The *x*-rays have been used in the treatment of a variety of different diseases, and there is little doubt that sometimes their use is decidedly beneficial—spleno-medullary leuchæmia being an instance in point. Latterly there have been advocates of the *x*-ray treatment of Graves' disease, and it is interesting to note the results obtained. In a disease whose course is so variable it is particularly difficult to be sure whether improvement in a patient's condition is really due to treatment or not. In the reports of many of the cases there is need to discount the optimism of the authors; on the other hand, many of the cases have been of the most difficult type, referred to the hospital *x*-ray departments as a last resource.

### The Prospects of Benefit.

As far as can be impartially judged, it seems clear that the *x*-rays are not especially liable to do harm to Graves' disease patients; that some cases are not benefited in the least; that fully-established cases are not likely to be cured, though amelioration is to be expected in the less severe cases; that the general nervousness and the tachycardia and the size of the neck are all more likely to improve than is the exophthalmos; and that in young patients who are developing Graves' disease, but in whom one or more of the classical symptoms are still wanting, complete cure is not unlikely.

Dr. C. Thurston Holland, of the *X*-Ray Department of the Liverpool Children's Infirmary, gives a very interesting and apparently unbiassed review of the subject, and of his own experiences in it, in the *Liverpool Medico-Chirurgical Journal*. He lays particular stress upon the greater likelihood of complete cure if the *x*-rays are used as soon as possible, either with or without medical treatment.

Thus a girl of fourteen came under observation with a pulse-rate of 120, a neck measurement of 13½ inches, but without exophthalmos or hand tremors. She was given fifty-two exposures of ten minutes each between March and November. She improved the whole time, and on October 14 the neck measurement was 13 inches and the pulse 108. By the following January she was to all intents and purposes quite well. Another girl of seventeen, with a history of two years' swelling of the neck, with a pulse of 108, extreme nervousness, and a tendency to profuse perspirations, but without tremors and without exophthalmos, had forty-one exposures of ten minutes each between January 22 and August 22. In this case the pulse of 108 at starting after three exposures was 86, and on April 18 was 76. The nervousness entirely disappeared.

### Dr. Holland's Conclusions.

Dr. Holland's general conclusions are as follows:

1. In nearly all the cases carefully noted there was an immediate drop in the pulse-rate following upon the first three or four exposures, and this in some of the cases was very noticeable. Further, the pulse-rate remained reduced.

2. The muscular tremors and general nervousness also almost always showed signs of improvement from the first, and continued to improve during the course of the treatment.

3. The circumference of the neck, taken over the most prominent part of the gland, in some cases diminished notably, whilst in others no diminution in size occurred. Perhaps what was more noticeable was that in cases where the gland had been tense and hard, and where there had been throbbing, it almost always became softer and less tense, and the throbbing in it diminished.

4. Exophthalmos was not materially altered when it was a marked feature. This is a very important point, because exophthalmos is often what troubles the patient most, and she may be particularly disappointed if she consents to the *x*-ray treatment, and after a long course is told that she is much better, and yet can observe little or no alteration in the state of her eyes. The absence of benefit to the exopthalmos is well expressed by Dr. Holland's words of disappointment: "In two or three we thought, and the patient's friends thought, there was some improvement, but at any rate it was small."

### Supposed Mode of Action of X-rays in Graves' Disease.

It was C. H. Mayo who noted in operating upon enlarged cervical glands that had been previously treated by frequent exposure to the *x*-rays that these glands were much sclerosed. It was with the idea of bringing about a similar sclerosis in the hypertrophied thyroid glands of patients suffering from various forms of goitre that the *x*-rays were first employed by him in these cases.

It is, however, exceedingly difficult to know what result one is bringing about when one applies such potent remedies as *x*-rays in what, notwithstanding all modern improvements, must still be regarded as a more or less rough-and-ready manner. If sclerosis of the thyroid gland is the object in view, how is one to say when sufficient sclerosis has been brought about? If too much sclerotic change ensues, myxœdema may develop in place of the former Graves' disease.

### The Danger of Myxœdema.

As a matter of fact, although it is well established that myxœdema may develop out of Graves' disease spontaneously, it does so only with the greatest rarity. Therefore, when one is told by Dr. Holland that "In Dr. Bruce's second case, when about 120 exposures of ten minutes each were given in one year, and where cure was reported (to the Royal Society of Medicine), the patient was shown and presented some symptoms suggestive of myxœdema. A friend of mine, who has also treated a few cases, has told me that one of his has also developed this condition" one realises that the *x*-rays do really cause enough sclerosis to convert Graves' disease into myxœdema. The change is not for the worse, of course, because myxœdema can be very successfully and simply remedied by giving thyroid extract daily by the mouth; but it would be preferable to stop the sclerosis of the thyroid gland before this result is produced, for which purpose it is recommended by Dr. Holland that in practice the *x*-rays should be applied only to one side of the gland to start with.

